# Safety and immunogenicity of a varicella vaccine without human serum albumin (HSA) versus a HSA-containing formulation administered in the second year of life: a phase III, double-blind, randomized study

**DOI:** 10.1186/s12887-019-1425-7

**Published:** 2019-02-07

**Authors:** Saul N. Faust, Maguelone Le Roy, Chitsanu Pancharoen, Miguel Angel Rodriguez Weber, Katrina Cathie, Ulrich Behre, Jolanta Bernatoniene, Matthew D. Snape, Klaus Helm, Carlos Eduardo Medina Pech, Ouzama Henry, Carmen Baccarini, Michael Povey, Paul Gillard

**Affiliations:** 1 NIHR Southampton Clinical Research Facility, University of Southampton and University Hospital Southampton NHS Foundation Trust, Tremona Road, Southampton, SO16 6YD UK; 2 GSK, Avenue Fleming 20, B-1300 Wavre, Belgium; 3 Department of Pediatrics and Center of Excellence for Pediatric Infectious Diseases and Vaccines, Faculty of Medicine, Chulalongkorn University, 1873 Rama 4 Road, Pathumwan, Bangkok, 10330 Thailand; 4 Instituto Nacional de Pediatria, Insurgentes Sur 3700C Col. Insurgentes Cuicuilco, Coyoacan, 04530 Mexico City, Mexico; 5 Private Practice, Hauptstrasse 240, 77694 Kehl, Germany; 6 Pediatric Infectious Disease Department, Education Centre Level 6, University Hospitals Bristol NHS Foundation Trust, Bristol Royal Hospital for Children, Upper Maudlin Street, Bristol, BS2 8AE UK; 7 Oxford Vaccine Group, Department of Pediatrics, University of Oxford and the NIHR Oxford Biomedical Research Centre, Headington, Oxford, OX3 9DU UK; 8 Private practice, Paulinenstrasse 71a, 32756 Detmold, Germany; 9 Medical Care and Research SA de CV, Calle 32 No. 217 Col. Garcia Gineres, 97070 Mérida, Yucatán Mexico; 10 GSK, 14200 Shady Grove Rd, Rockville, MD 20850 USA; 11 GSK at the time of study conduct, 160 North Gulph Road, King of Prussia, PA 19406 USA

**Keywords:** Varicella vaccine, Safety, Non-inferiority, Human serum albumin

## Abstract

**Background:**

A new formulation of the live-attenuated varicella vaccine *Varilrix* (GSK) produced without human serum albumin (HSA) was developed to minimize a theoretical risk of transmission of infectious diseases. A previous study showed that the vaccine was immunologically non-inferior to the HSA-containing vaccine and well-tolerated in toddlers; low-grade fever was numerically higher in children receiving the vaccine without HSA, but the study lacked power to conclude on this difference.

**Methods:**

In this phase III, double-blind, multi-center study, healthy 12–23-month-olds were randomized (1:1) to receive two doses of the varicella vaccine without (Var-HSA group) or with HSA (Var + HSA group) at days 0 and 42. The primary objective compared safety of the vaccines in terms of incidence of fever > 39.0 °C in the 15-day period post-first vaccination. The objective was considered met if the upper limit of the 95% confidence interval for the between-group difference in the incidence of fever > 39.0 °C was ≤5% (Var-HSA group minus Var + HSA group). Safety, reactogenicity and immune responses were evaluated.

**Results:**

Six hundred fifteen children in the Var-HSA group and 616 in the Var + HSA group received ≥1 vaccination. Fever > 39.0 °C was reported in 3.9 and 5.2% of participants in the Var-HSA and Var + HSA groups, with a between-group difference of − 1.29 (95% confidence interval: − 3.72–1.08); therefore, the primary objective was achieved. Fever rates post-each dose and the incidence of solicited local and general adverse events (AEs) were comparable between groups. Unsolicited AEs were reported for 43.9 and 36.5% of children in the Var-HSA group and 45.8 and 36.0% of children in the Var + HSA group, during 43 days post-dose 1 and 2, respectively. Serious AEs occurred in 2.1% (group Var-HSA) and 2.4% (group Var + HSA) of children, throughout the study. In a sub-cohort of 364 children, all had anti-varicella-zoster virus antibody concentrations ≥50 mIU/mL post-dose 2; comparable geometric mean concentrations were observed between the groups.

**Conclusions:**

The varicella vaccine formulated without HSA did not induce higher rates of fever during the 15 day-post-vaccination period, as compared with the original HSA-containing vaccine. The two vaccines displayed similar safety and immunogenicity profiles in toddlers.

**Trial registration:**

NCT02570126, registered on 5 October 2015 (www.clinicaltrials.gov).

**Electronic supplementary material:**

The online version of this article (10.1186/s12887-019-1425-7) contains supplementary material, which is available to authorized users.

## Introduction

The burden of disease for varicella remains important, with conservative estimates of 4.2 million severe complications leading to hospitalization and 4200 deaths occurring globally each year [1]. Despite the fact that the disease is vaccine preventable with vaccination being highly effective, not all countries recommend routine immunization [2].

The live attenuated varicella vaccine *Varilrix* (GSK) has been successfully used in routine vaccination programs [3]. The original formulation of the vaccine contains human serum albumin (HSA). Historically, HSA was an excipient frequently added at vaccine formulation to improve the stability of lyophilised live attenuated vaccines. Even if HSA has excellent clinical safety records [4], the use of human plasma-derived products in the manufacture of biologicals is associated with a theoretical risk of contamination with adventitious agents and the subsequent potential transmission of infectious diseases [5]. Therefore, in line with recommendations of the European Medicines Agency [5], a new formulation of the varicella vaccine *Varilrix* does not include HSA, while ensuring equivalent stability. Currently, both formulations are approved for use worldwide, with the new formulation without HSA being already authorized in several European countries, Canada, Australia and New Zealand.

In a previous study in children 11–21 months of age conducted in two European countries, the immunogenicity of a first dose of varicella vaccine without HSA was demonstrated to be non-inferior to that of the HSA-containing varicella vaccine and both formulations showed acceptable safety profiles [6]. Unexpectedly, after the first vaccination, a slightly higher rate of fever ≥37.5 °C, but not in fever > 39.0 °C, was observed in children receiving the vaccine produced without HSA (28.1–95% CI: 20.3–37.0%) as compared to the HSA-containing varicella vaccine (18.0–95% CI: 11.7–26.0%) [6].

This study was conducted to confirm, in 1-year old children, the safety profile of the varicella vaccine produced without HSA compared to the HSA-containing vaccine specifically in terms of severe (grade 3) fever, given the previously observed difference in the occurrence of fever.

**Fig. 1:**
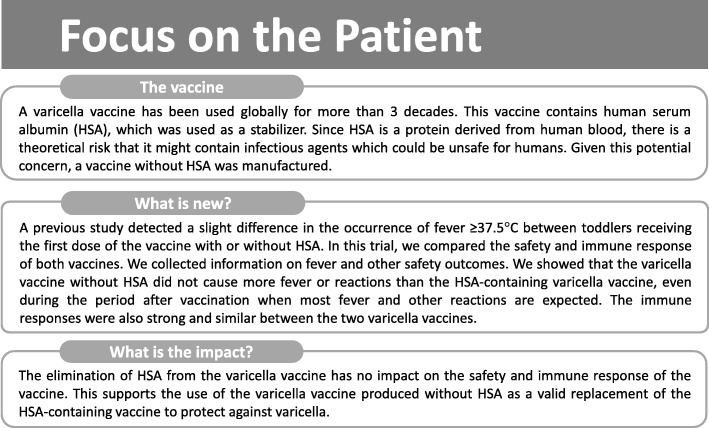
Focus on the patient

A summary of the clinical relevance of the research, aimed to be shared with patients by health care providers, is represented in Fig. 1.

## Methods

### Study design and participants

This phase III, double-blind, randomized study was conducted in 5 countries (Estonia, Germany, Mexico, Thailand, and the United Kingdom) between November 2015 and October 2016. Healthy children aged 12–23 months were randomized (1:1) to receive either the varicella vaccine produced without HSA (Var-HSA group) or the HSA-containing varicella vaccine (Var + HSA group) administered as a 2-dose schedule at days 0 and 42.

Children were included in the study if they had a prior dose of measles, mumps and rubella vaccine at least 30 days prior to first study vaccination and if the investigator believed that compliance with the protocol requirements would be achieved. Children with any history of varicella disease, vaccination against varicella, recent varicella or zoster exposure (within 30 days prior to the study) were excluded from the study.

Randomization was performed accounting for country, center and immunogenicity sub-cohort, using a central, internet-hosted randomization system.

The vaccines were presented as lyophilized pellets, which were reconstituted with 0.5 mL of water for injection, before subcutaneous administration in the left upper arm. Both vaccines contained live attenuated varicella virus (Oka/RIT strain) with a potency of ≥10^3.3^ plaque-forming units per dose.

Written informed consent was obtained from each parent/legally acceptable representative before vaccination. The study was conducted in accordance with Good Clinical Practice and the Declaration of Helsinki and is registered at www.Clinicaltrials.gov (NCT02570126). A summary of the protocol is available at https://www.gsk-studyregister.com (study ID 200147).

### Study objectives

The primary objective of the study was to demonstrate the absence of increased rates of fever > 39.0 °C in the 15-day period following the first vaccination, for the varicella vaccine without HSA when compared with the HSA-containing varicella vaccine. The success criterion was set at 5% for the upper limit of the 2-sided standardized asymptotic 95% confidence interval (CI) of the difference (vaccine without HSA group minus HSA-containing varicella vaccine group).

Secondary objectives assessed the safety and reactogenicity of both vaccines and the incidence of fever ≥38.0 °C after each vaccination, including fever temporally-associated with serious adverse events (SAEs). Immune responses were evaluated in terms of seroresponse rates and geometric mean concentrations (GMCs) for anti-varicella-zoster virus (VZV) antibodies in a sub-cohort of participants, at 43 days post-each dose.

### Safety and reactogenicity assessment

Solicited local AEs (pain, redness and swelling at injection site) were recorded for 4 days (days 0–3), while solicited general AEs (varicella-like rash, fever [axillary temperature ≥ 38.0 °C], other rashes and febrile convulsions) and unsolicited AEs were followed-up for 43 days (days 0–42) post-each vaccination. Fever starting within 7 days from an SAE was considered temporally-associated with the event and was recorded for 15 days (days 0–14) from each dose. SAEs were recorded throughout the study.

The intensity of each solicited and unsolicited AE was graded by the investigator from mild to severe, based on measurements recorded by the parents/legally acceptable representatives on diary cards. All reported varicella-like rashes were evaluated and confirmed by the investigator. Any suspected febrile convulsions were classified using the Brighton Collaboration levels of diagnostic certainty [7] and the American Academy of Pediatrics definitions [8]. All solicited local AEs were considered as causally-related to vaccination; the causality of all other AEs was assessed by the investigator.

### Immunogenicity assessment

Blood samples were collected from sub-cohorts of participants (± first 400 subjects, after randomization to the treatment groups) pre-vaccination and at 42 days post-dose 1 vaccination and 42 days post-dose 2 vaccination. Anti-VZV immunoglobulin G concentrations were measured using a commercial enzyme linked immunosorbent assay (*Enzygnost*, Dade Behring, Marburg, Germany) with a cut-off of 25 m-International Units [mIU]/mL. Seroresponse was defined as post-vaccination anti-VZV antibody concentration ≥ 50 mIU/mL for participants who were seronegative (antibody concentration < 25 mIU/mL) before vaccination. Anti-VZV antibody levels ≥50 mIU/mL were considered to offer clinical benefit.

### Statistical analyses

A sample size of 1220 participants was calculated to reach at least 579 evaluable participants in each group. The power to meet the statistical criterion under the null hypothesis of no vaccine difference and a one-sided type 1 error of 2.5% was 96.3%.

The safety analysis was performed on the total vaccinated cohort, including all participants receiving at least one study vaccine dose.

**Fig. 2:**
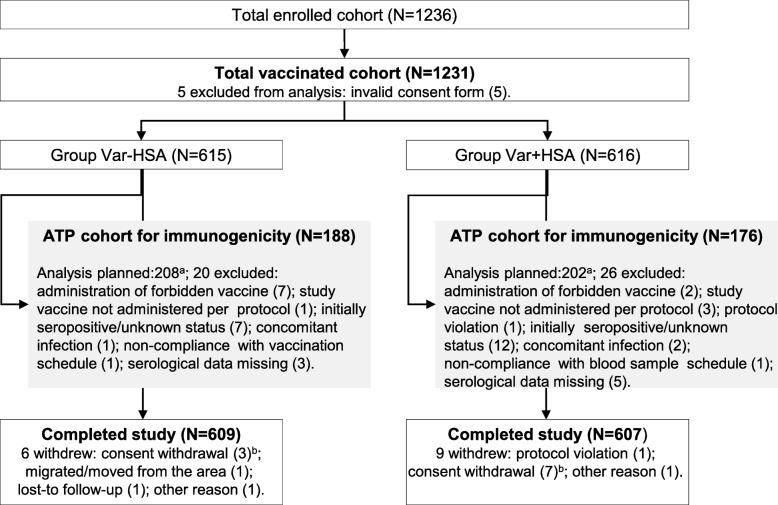
Participant flow chart. Group Var-HSA, participants receiving varicella vaccine produced without HSA (human serum albumin); Group Var + HSA, participants receiving varicella vaccine containing HSA; N, number of participants; ATP, according-to-protocol. Note: **a** Immunogenicity analyses were only planned in a sub-cohort of ~ 200 participants in each group. **b** Not due to an adverse event

The immunogenicity analysis was carried out on sub-cohorts of ~ 200 participants from each group, who were included in the according-to-protocol (ATP) cohort for immunogenicity. The ATP cohort consisted of eligible participants (see exclusion criteria in Fig. 2) with available results who were seronegative at pre-vaccination. Anti-VZV antibody GMCs were computed by taking the antilog of the mean of the log concentration transformations of all values ≥40 mIU/mL (the lower limit of quantitation), with anti-VZV antibody concentrations of ≥40 mIU/mL–25 mIU/mL being given a value of 25 mIU/mL and concentrations below the assay cut-off being given an arbitrary value of 12.5 mIU/mL.

## Results

### Demographics

A total of 1231 participants were vaccinated and 1216 completed the study; the reasons for withdrawal from the study are indicated in Fig. 2. The ATP cohort for immunogenicity included 364 participants: 188 in the Var-HSA group and 176 in the Var + HSA group (Fig. 2).

The majority of participants (≥61.6%) were White-Caucasian. The demographic characteristics were similar between the two vaccine groups (Table 1).

**Table 1:** Demographic characteristics (total vaccinated cohort)

	Group Var-HSA (*N* = 615)	Group Var + HSA (*N* = 616)
Mean age at first vaccination (SD), months	16.7 (3.3)	16.9 (3.4)
Female, n (%)	318 (51.7)	312 (50.6)
Country of enrolment, *n* (%)		
Estonia	82 (13.3)	82 (13.3)
Germany	119 (19.3)	118 (19.2)
Mexico	71 (11.5)	71 (11.5)
Thailand	133 (21.6)	132 (21.4)
United Kingdom	210 (34.1)	213 (34.6)
Geographic ancestry, n (%)		
African/ African American heritage	3 (0.5)	1 (0.2)
Asian-Central South Asian heritage	2 (0.3)	2 (0.3)
Asian-East Asian heritage	3 (0.5)	2 (0.3)
Asian-South East Asian heritage	133 (21.6)	135 (21.9)
White-Caucasian/European heritage	379 (61.6)	381 (61.9)
Other	95 (15.5)	95 (15.4)
Ethnicity, *n* (%)		
Hispanic or Latino	75 (12.2)	73 (11.9)
Not Hispanic or Latino	540 (87.8)	543 (88.1)

Group Var-HSA, participants receiving varicella vaccine produced without HSA (human albumin serum); Group Var + HSA, participants receiving varicella vaccine containing HSA; *N* number of participants in each group, *SD* standard deviation; *n* (%), number (percentage) of participants in each category

### Safety and reactogenicity

In the 15-day period post-dose 1, fever > 39.0 °C was reported for 3.9% children in the Var-HSA group and 5.2% of children in the Var + HSA group. The between-group difference was − 1.29 (95% CI: -3.72–1.08), hence the primary objective of the study was achieved. In exploratory analyses, a between-group comparison showed similar rates of fever between 38.0 and 40.0 °C, with increments of 0.5 °C, reported during the 15-day period post dose-1 (Table 2).

**Table 2:** Incidence of fever reported during the 15-day (days 0–14) and results of between-group exploratory analyses, post-dose 1 (total vaccinated cohort)

	*n* (%)		Difference in percentage (Var-HSA minus Var + HSA), % (95% CI)
	Group Var-HSA	Group Var + HSA	
Post-dose 1	*N* = 612	*N* = 614	
Any	83 (13.6)	92 (15.0)	−1.42 (−5.36–2.51)
≥38.0 °C	83 (13.6)	92 (15.0)	−1.42 (−5.36–2.51)
related	53 (8.7)	33 (5.4)	3.29 (0.43–6.23)
> 38.5 °C	42 (6.9)	45 (7.3)	−0.47 (−3.39–2.45)
related	21 (3.4)	14 (2.3)	1.15 (−0.75–3.15)
> 39.0 °C	**24 (3.9)**	**32 (5.2)**	**−1.29 (−3.72–1.08)**
related	12 (2)	10 (1.6)	0.33 (−1.25–1.96)
> 39.5 °C	18 (2.9)	13 (2.1)	0.82 (−0.99–2.71)
related	9 (1.5)	1 (0.2)	1.31 (0.38–2.63)
> 40.0 °C	5 (0.8)	5 (0.8)	0 (−1.17–1.18)
related	1 (0.2)	0 (0.0)	0.16 (−0.46–0.92)
Medical advice	19 (3.1)	28 (4.6)	−1.46 (−3.72–0.72)

Group Var-HSA, participants receiving varicella vaccine produced without HSA (human albumin serum); Group Var + HSA, participants receiving varicella vaccine containing HSA; *n* (%), number (percentage) of participants reporting the symptom at least once, *CI* confidence interval; *N* number of participants with available results

Note: Bolded values indicate that the primary objective was achieved (the upper limit of the 95% CI ≤5% for the between-group difference in incidence of fever > 39.0 °C). All other comparisons were exploratory, without adjustment for multiplicity; therefore, the results should be interpreted with caution

Any fever (temperature ≥ 38.0 °C) was reported for 13.6% of children in the Var-HSA group following each dose and for 15.0% (post-dose 1) and 14.1% (post-dose 2) of children in the Var + HSA group (Table 2 and Additional file 1: Table S1). The onset, duration and outcome of the reported fever cases were similar between the two vaccine groups (data not shown).

In the Var-HSA group, fever ≥38.0 °C was temporally-associated with a SAE (acute bronchitis and acute nasopharyngitis, post-dose 1 and pneumonia, post-dose 2) for 0.3% (95% CI: 0.0–1.2) and 0.2% (95% CI: 0.0–0.9) of children, respectively. In the Var + HSA group, temporally-associated fever was reported for 0.3% (95% CI: 0.0–1.2) of children following each dose, with the SAEs being acute serious otitis media and worsening bronchitis, post-dose 1 and pseudocroup and otitis, post-dose 2. None of these events were considered as causally related to the vaccination by the investigator.

**Fig. 3:**
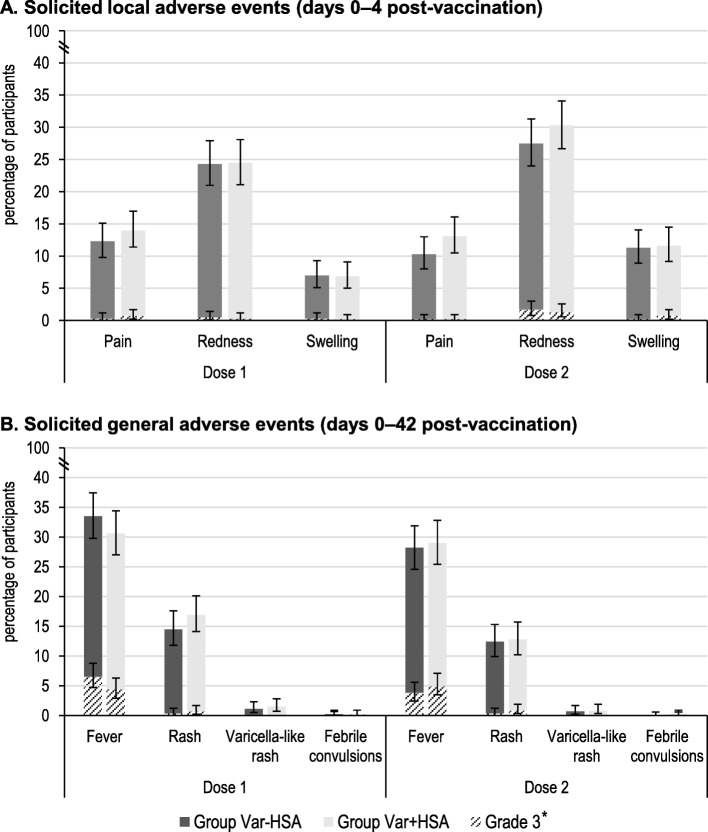
Percentage of participants with solicited local and general adverse events, post-each dose (total vaccinated cohort). Group Var-HSA, participants receiving varicella vaccine produced without HSA (human albumin serum); Group Var + HSA, participants receiving varicella vaccine containing HSA. Note: Error bars represent 95% confidence intervals. Grade 3 adverse events were defined as: cried when limb was moved/spontaneously painful for pain; diameter > 20 mm for swelling/redness; temperature > 39.5 °C for fever; > 150 lesions for varicella-like rash; prevented normal, everyday activities and leading to seeking medical advice (all other events). *Two grade 3 varicella-like rashes were reported in this study, both of which were following dose 1 in the Var + HSA group

The most common solicited local AE reported after each vaccination was redness, in 24.3% (post-dose 1) and 27.5% (post-dose 2) of children in the Var-HSA group, compared to 24.5% (post-dose 1) and 30.3% (post-dose 2) of children in the Var + HSA group. Grade 3 solicited local AEs were rare (Fig. 3a). Any rash was reported in 14.5 and 12.4% of participants in the Var-HSA group and in 16.9 and 12.8% of children in the Var + HSA group, post-dose 1 and 2, respectively. In both vaccine groups, following each vaccination, varicella-like rash was reported in < 1.5% of children (Fig. 3b). Febrile convulsion was reported in a low percentage of children (0.2% in each group post-dose 1 and 0.2% in the Var + HSA group, post-dose 2) (Fig. 3b). All these cases occurred with concurrent AEs, were recovered/resolved before study end and none of them were considered vaccination-related by the investigator.

At least one unsolicited AE was reported for 43.9 and 36.5% of children in the Var-HSA group and in 45.8 and 36.0% of children in the Var + HSA group, post-dose 1 and 2, respectively. In both vaccine groups, nasopharyngitis was the most common unsolicited AE, reported for 9.1–9.6% and 6.2–6.5% of children post-dose 1 and 2, respectively.

SAEs were recorded for 2.1 and 2.4% of children in the Var-HSA and Var + HSA groups, respectively. All SAEs were resolved before study end, none were considered as related to study vaccination, and none were fatal.

### Immunogenicity

At 43 days post-dose 1, seroresponse was observed for 94.1% of children in the Var-HSA group and 98.8% of those in the Var + HSA group. All children had anti-VZV antibody concentrations ≥50 mIU/mL post-dose 2. Antibody GMCs were comparable between vaccine groups at both time points (Table 3).

**Table 3:** Immunogenicity results at 43 days post-each dose (according-to-protocol cohort for immunogenicity)

	Group Var-HSA			Group Var+HSA		
	N	SRR (95% CI), %	GMC (95% CI), mIU/mL	N	SRR (95% CI), %	GMC (95% CI), mIU/mL
Post-dose 1	185	94.1 (89.6–97.0)	139.9 (126.7–154.5)	168	98.8 (95.8–99.9)	146.0 (132.5–160.7)
Post-dose 2	180	100 (98.0–100)	931.8 (841.1–1032.3)	173	100 (97.9–100)	1102.4 (996.1–1220.2)

*Group Var-HSA* participants receiving varicella vaccine produced without HSA (human albumin serum), *Group Var+HSA* participants receiving varicella vaccine containing HSA, *N* number of participants with available results, *SRR* seroresponse rate, *CI* confidence interval, *GMC* geometric mean concentration, *IU* international units

Note: Seroresponse was defined as post-vaccination anti-varicella-zoster virus antibody concentrations ≥50 mIU/mL

## Discussion

Our results showed that the incidence of fever > 39.0 °C following the administration of one dose of varicella vaccine produced without HSA was not significantly different from that observed after vaccination with the HSA-containing varicella vaccine, which was the primary objective of this trial. The 39.0 °C threshold in the present study was selected based on its clinical significance in the targeted age group, in view of the potential consequences in terms of need for medical advice, complications, or hospitalizations [9].

A previous trial indicated a slight numerical increase in the incidence of fever ≥37.5 °C for the varicella vaccine without HSA, but the statistical significance of the difference could not be evaluated, as the study was not powered to detect differences in terms of fever between groups [6]. Our study included a larger number of children of the same age (1231 vs 244 in the previous study) and was powered to assess any statistically-significant increase in rate of fever > 39.0 °C in the 15-day period post-first vaccination.

In the current study, although a slight increase in the incidence of fever > 38.0 °C related to vaccination was observed for the varicella vaccine without HSA, exploratory analyses showed that the incidence of fever of any severity was similar between the two groups in the 15-day period following first vaccination. Similar fever rates were found for low grade fever (> 38.0 °C) between the two vaccine groups for the 8-day post-first vaccination, in contrast with previous observations [6]. No increase in the incidence of fever was observed after the second dose and data related to the intensity, onset, duration and outcome of the reported fever cases post-each vaccination did not indicate any clinically significant difference between the two vaccines. Moreover, fever ≥38.0 °C associated with an SAE was uncommon in both groups, and none of the reported SAEs were considered as related to vaccination.

The incidence of solicited and unsolicited AEs were comparable between groups and consistent with the previous reports for the HSA-containing varicella vaccine, which has shown an acceptable safety profile in clinical trials and post-marketing studies [3, 4, 10]. Mild varicella-like rash is usually observed in < 5% of children aged between 12 months and 12 years, following vaccination with a varicella vaccine [11, 12], while in our study, varicella-like rashes were reported in ≤1.5% of participants in both groups. It is worth noting that molecular analysis performed on the varicella cases among vaccinated children in the present study revealed that such patients were infected with the wild type virus. However, the incidence of reported varicella-like rashes following administration of the HSA-containing vaccine was shown to vary, from 0.0% in Taiwanese children 15–18 months old receiving a measles-mumps-rubella vaccine concomitantly with the varicella vaccine [13] to up to 6.4% of children 1–12 years of age in a Canadian study [14]. Overall, in the Var-HSA group, the percentage of participants with both solicited and unsolicited AEs was similar or even lower following the second vaccination compared to the first vaccine dose. No safety concerns were identified during the study.

The immunogenicity of the two vaccines following each vaccination was comparable, in line with previously reported results [6]. Following first vaccination, point-estimates for seroresponse rates were lower in the group receiving the vaccine without HSA, but the 95% CIs were overlapping and the study was not powered to assess any statistical difference for immunogenicity results. This difference is not likely to be clinically significant, seeing that all children in both groups showed seroresponse following the second dose.

The main strengths of our study were the large sample size which allowed the detection of statistically significant differences for fever > 39.0 °C; and the study conduct across countries in three different continents. Also, the safety data collected in parallel to fever allowed the evaluation of the clinical significance.

Our study had some limitations. Although the study was conducted in children from various geographical regions, most children were White-Caucasian, due to the higher number of participants recruited from European countries; therefore, the generalization of the results to larger populations might be somewhat hindered. The exploratory analyses were performed without adjustment for multiplicity and therefore, their results should be interpreted with caution.

## Conclusions

A new formulation of the varicella vaccine produced without HSA was developed to minimize the theoretical risks of contamination. The vaccine was not associated with an increased incidence of post-vaccination fever as compared with the historical HSA-containing varicella vaccine. The safety and immunogenicity profiles of the two vaccines were clinically acceptable and comparable when administered as a 2-dose regimen in children 12–23 months old.

## Additional file

Additional file 1: Incidence of fever reported during different follow-up periods post-vaccination. **Table S1**. Incidence of fever reported during the 15-day (days 0–14) post-vaccination period, post-dose 2 (total vaccinated cohort). **Table S2**. Incidence of fever reported during the 8-day period (days 0–7) post-vaccination period (total vaccinated cohort). (DOCX 15 kb)

## Abbreviations

AE: Adverse event
ATP: According-to-protocol
CI: Confidence interval
GMC: Geometric mean concentration
HSA: Human serum albumin
IU: International Units
SAE: Serious adverse event
VZV: Varicella-zoster virus
