# Supplementary material for: Safety and immunogenicity of a varicella vaccine without human serum albumin (HSA) versus a HSA-containing formulation administered in the second year of life: a phase III, double-blind, randomized study
Source: BMC Pediatr. 2019 Feb 7;19:50. doi: 10.1186/s12887-019-1425-7 (PMC6366055; doi:10.1186/s12887-019-1425-7)
Supplement: Supplementary file 1 — Incidence of fever reported during different follow-up periods post-vaccination. Table S1. Incidence of fever reported during the 15-day (days 0–14) post-vaccination period, post-dose 2 (total vaccinated cohort). Table S2. Incidence of fever reported during the 8-day period (days 0–7) post-vaccination period (total vaccinated cohort). (DOCX 15 kb) [file 12887_2019_1425_MOESM1_ESM.docx]

# Additional file: Incidence of fever reported during different follow-up periods post-vaccination

# Table A1_1. Incidence of fever reported during the 15-day (days 0–14) post-vaccination period, post-dose 2 (total vaccinated cohort)

|  | % (95% CI) | | |
| --- | --- | --- | --- |
|  | Group Var-HSA  (N=611) |  | Group Var+HSA  (N=610) |
| Any | 13.6 (11–16.6) |  | 14.1 (11.4–17.1) |
| ≥38.0 °C | 13.6 (11–16.6) |  | 14.1 (11.4–17.1) |
| related | 6.5 (4.7–8.8) |  | 7.4 (5.4–9.7) |
| >38.5 °C | 6.5 (4.7–8.8) |  | 6.9 (5.0–9.2) |
| related | 2.6 (1.5–4.2) |  | 3.3 (2.0–5.0) |
| >39.0 °C | 2.9 (1.8–4.6) |  | 4.1 (2.7–6.0) |
| related | 1.0 (0.4–2.1) |  | 1.6 (0.8–3.0) |
| >39.5 °C | 0.7 (0.2–1.7) |  | 2.3 (1.3–3.8) |
| related | 0.3 (0.0–1.2) |  | 1.1 (0.5–2.4) |
| >40.0 °C | 0.3 (0.0–1.2) |  | 0.5 (0.1–1.4) |
| related | 0.2 (0.0–0.9) |  | 0.3 (0.0–1.2) |
| Medical advice | 4.1 (2.7–6.0) |  | 4.3 (2.8–6.2) |

%, percentage of participants of participants reporting the symptom at least once; CI, confidence interval; Group Var-HSA, participants receiving varicella vaccine produced without HSA (human albumin serum); Group Var+HSA, participants receiving varicella vaccine containing HSA; N, number of participants with available results.

# Table A1_2. Incidence of fever (any and related to vaccination) reported during the 8-day (days 0–7) post-vaccination period (total vaccinated cohort)

|  | % (95% CI) | | | | |
| --- | --- | --- | --- | --- | --- |
|  | Post-dose 1 | |  | Post-dose 2 | |
|  | Group Var-HSA (N=612) | Group Var+HSA  (N=614) |  | Group Var-HSA (N=611) | Group Var+HSA (N=610) |
| Any | 9.5 (7.3–12.1) | 9.1 (7–11.7) |  | 9.2 (7–11.7) | 8.7 (6.6–11.2) |
| ≥38.0 °C | 9.5 (7.3–12.1) | 9.1 (7–11.7) |  | 9.2 (7–11.7) | 8.7 (6.6–11.2) |
| related | 6.4 (4.6–8.6) | 3.7 (2.4–5.6) |  | 4.4 (2.9–6.4) | 4.9 (3.3–6.9) |
| >38.5 °C | 4.9 (3.3–6.9) | 4.1 (2.7–6.0) |  | 3.6 (2.3–5.4) | 4.3 (2.8–6.2) |
| related | 2.8 (1.6–4.4) | 1.5 (0.7–2.8) |  | 1.6 (0.8–3.0) | 2.3 (1.3–3.8) |
| >39.0 °C | 2.6 (1.5–4.2) | 2.8 (1.6–4.4) |  | 1.5 (0.7–2.8) | 2.5 (1.4–4.0) |
| related | 1.5 (0.7–2.8) | 1.0 (0.4–2.1) |  | 0.7 (0.2–1.7) | 1.3 (0.6–2.6) |
| >39.5 °C | 1.6 (0.8–3.0) | 0.8 (0.3–1.9) |  | 0.7 (0.2–1.7) | 1.3 (0.6–2.6) |
| related | 1.0 (0.4–2.1) | 0.2 (0–0.9) |  | 0.3 (0.0–1.2) | 0.8 (0.3–1.9) |
| >40.0 °C | 0.5 (0.1–1.4) | 0.3 (0.0–1.2) |  | 0.2 (0.0–0.9) | 0.2 (0.0–0.9) |
| related | 0.2 (0.0–0.9) | 0.0 (0.0–0.6) |  | 0.2 (0.0–0.9) | 0.0 (0.0–0.6) |
| Medical advice | 1.8 (0.9–3.2) | 1.6 (0.8–3.0) |  | 2.5 (1.4–4.0) | 2.1 (1.1–3.6) |

%, percentage of participants reporting the symptom at least once; CI, confidence interval; Group Var-HSA, participants receiving varicella vaccine produced without HSA (human albumin serum); Group Var+HSA, participants receiving varicella vaccine containing HSA; N, number of participants with available results.
